# Aspartic protease supplementation enhancing the performance, carcass characteristics, nutrient digestibility and economic viability, without changing blood parameters and salivary cortisol of pigs

**DOI:** 10.1038/s41598-024-62006-1

**Published:** 2024-05-16

**Authors:** Thiago Augusto da Cruz, Bruno Bracco Donatelli Muro, Eduardo Machado Costa Lima, Valéria dos Santos Moreira, Julio Cesar Carrera de Carvalho, Cesar Augusto Pospissil Garbossa, Leandro Batista Costa

**Affiliations:** 1 School of Veterinary Medicine and Animal Science, University of São Paulo, Av. Duque de Caxias Norte, 225, Pirassununga, SP 13635-900 Brazil; 2 Mcassab Indústria e Comércio, Av. das Nações Unidas, 20.882, São Paulo, SP 04795-000 Brazil; 3 Present Address: Graduate Program in Animal Science, School of Medicine and Life Sciences, Pontifical Catholic University of Paraná, St. Imaculada Conceição, 1155, Curitiba, PR 80215-901 Brazil

**Keywords:** Animal welfare, Enzymes, Hematological analyzes, Protein, Swine, Proteases, Enzymes, Animal physiology

## Abstract

Aiming to study the performance, carcass characteristics, nutrient digestibility, blood parameters, salivary cortisol levels, and economic viability of pigs administered aspartic protease, a total of 135 pigs were housed in pens in a randomized block design, divided into five treatments with nine replications. The experimental diets were positive control (PC), basic diet with a 5.0% reduction in protein and amino acid requirements; negative control (NC) with a 7.5% reduction in protein and amino acid requirements; NC + 100 g/mT of aspartic protease (NC100); NC + 150 g/mT of aspartic protease (NC150); and NC + 200 g/mT of neutral serine protease (NC200). The inclusion of protease, independently of the source and amount, increased the average daily weight gain (*P* < 0.05) of animals compared with the control treatments (PC and NC), improved feed conversion (*P* < 0.05) in early stages, and improved diet digestibility (*P* < 0.05) compared with the PC. Treatment with NC150 and NC200 resulted in greater carcass weights (*P* < 0.05) than treatment with the PC. NC100 led to a greater carcass yield than PC (*P* < 0.05), and NC150 resulted in a greater loin eye area than PC (*P* < 0.05). No differences (*P* > 0.05) in the blood parameters or salivary cortisol levels were found. Regarding economic viability, proteases increased the profitability, with NC150 leading to the best results. Thus, the use of aspartic proteases is recommended to improve performance and further facilitate pork production.

## Introduction

Exogenous enzymes have been added to the diets of swine for several decades. The inclusion of such enzyme is primarily due to the availability of nutrients whose endogenous enzymes cannot hydrolyze, for instance, non-starch polysaccharides using carbohydrase; inactivation of antinutritional factors, such as phytate by phytase; supplementation of endogenous enzymes, such as amylase and proteases^1^; and the economic gain derived from the inclusion of this technology^2^. The most used enzymes in animal diets, in order of importance, are phytase and carbohydrase^3^, which have well-defined actions; however, the findings regarding the use of proteases are quite divergent^4^ and there as limited information on the effect of including proteases alone in pig diets^5^.

Similar to all enzymes, proteases require an aqueous medium, adequate pH and temperature, and specific substrates to act efficiently^6^; however, unlike other exogenous enzymes, proteases have complex specificities. In general, proteases are classified according to their medium of action, becoming active at different pH values. The site of action of the protein molecule is divided between endoproteases and exoproteases. Endoproteases perform hydrolysis in the middle of the peptide chain, while exoproteases perform hydrolysis at the end of the peptide chain^7^.

Proteases can be divided into four main classes based on their catalytic mechanisms: serine proteases, cysteine proteases; aspartic proteases; and metalloproteases. This classification is based on the nature of the amino acid residue, which acts as a nucleophile in the active site of the protease and is widely used in the fields of biochemistry and molecular biology^8^.

In animal metabolism is possible to find all four types of proteases, in the case of aspartic and serine proteases, we have pepsin and trypsin, respectively^9^. Aspartic proteases are characterized by the presence of two catalytic aspartate residues at its active site, and is predominantly found in the stomach, where it plays a crucial role in the initial stages of protein digestion and exhibits broad specificity, cleaving peptide bonds adjacent to hydrophobic amino acids such as phenylalanine, tryptophan, and leucine^9^. On the other hand, serine protease, meaning it contains a serine residue at its active site, which is crucial for its catalytic activity. It typically consists of a catalytic triad composed of serine, histidine, and aspartate residues. Its primarily functions in the small intestine, where it catalyzes the hydrolysis of peptide bonds in proteins and polypeptides. It cleaves peptide bonds on the carboxyl side of lysine and arginine residues^9^.

Another relevant point is that exogenous enzymes are added to pig diets using a nutritional matrix. As a result, there is normally a decrease in the amounts of protein and amino acids. The aim of this is to reduce animal feed costs, however, dietary proteins and amino acids directly affect swine health^10^ and welfare. Hence, increasing protein digestibility using exogenous enzymes could change blood parameters and salivary cortisol levels, and improve swine performance, because excessive protein intake significantly increases salivary cortisol concentration in pigs^11^.

The hypothesis posits that the reduction in protein and amino acids (NC) will yield inferior outcomes, with no discernible difference observed among the enzyme treatments (NC100, NC150, and NC200) and the positive control (PC).

The objective of this study was to evaluate the effects of acid aspartic protease (EC 3.4.23.18) and another neutral serine protease (EC 3.4.21.15) as a benchmark, on the performance, carcass characteristics, diet digestibility, blood parameters, salivary cortisol levels, and economic return of rearing pigs in the growing and finishing phases.

## Materials and methods

### Animal ethics statement

All procedures involving the animals were approved by the Ethics Committee in Use of Animals of the School of Veterinary Medicine and Animal Science, University of São Paulo (approval number, CEUA 8428221220). We confirm that all experiments were performed in accordance with relevant guidelines and regulations in animal research: Reporting of In Vivo Experiments guidelines (ARRIVE guidelines (https://arriveguidelines.org)) were followed for the in-vivo studies.

The assay was performed at the experimental farm of the Swine Research Laboratory (SRL) of the Department of Animal Nutrition and Production of the School of Veterinary Medicine and Animal Science at the University of São Paulo (USP), located at the USP Fernando Costa Campus, Pirassununga, São Paulo. The study was conducted between November 2020 and March 2021.

### Animals

A total of 135 piglets, 75 barrows, and 60 gilts were obtained from a commercial swine herd. At the start of the experiment, the animals were 63 ± 1 days old and had an average weight of 25.56 ± 0.04 kg. The animals were housed in pens in the growth and finishing of the SRL. The pens had a compact floor with a shallow pool (used in all pens to help control the temperature), semi-automatic feeders, nipple drinkers, and the capacity to house four animals.

### Experimental design

The experimental design consisted of randomized blocks (initial weight and sex) with five treatments and nine replicates per treatment. Eight replicates comprised pens with three animals, and one replicate comprised pen with two animals. The experimental unit was defined as an average pen (two or three animals), except for the analyses performed after slaughter, in which each animal was considered an experimental unit. The experimental period was 104 days and was divided into four periods accordingly to feed changes and nutritional phases: growth 1, 1–26 days; growth 2, 27–49 days; finishing 1, 50–75 days; and finishing 2, 76–104 days^12^.

### Experimental diets and treatments

The diets were provided by MCassab Indústria and Comércio, Brazil (Table 1). The treatments were: positive control (PC), according to the recommendations of Rostagno et al.^12^, with a 5.0% reduction in protein and amino acid requirements; negative control (NC), also following the recommendations of Rostagno et al.^12^, with a 7.5% reduction in protein and amino acid requirements; NC + 100 g/mT of aspartic protease (NC100); NC + 150 g/mT of aspartic protease (NC150); and NC + 200 g/mT of serine protease EC 3.4.23.18 (NC200). Weight equivalent to kaolin was removed to include this enzyme. The strategy of using a 5% reduction in crude protein and amino acids for PC aimed to create a nutritional challenge. This was proposed because the animals were not subjected to dietary restriction and therefore, it was assumed that the high consumption by the animals would make it impossible to observe the effects of the enzymes. Thus, the NC assumed a reduction of 2.5% in relation to the PC, assuming this amount as the supposed capacity for nutritional release by the enzymes.

**Table 1:** Formulation and proximate composition of the experimental diets (kg/mT).

Ingredients	PC—Positive control				NC—Negative control			
	Growth 1	Growth 2	Finishing 1	Finishing 2	Growth 1	Growth 2	Finishing 1	Finishing 2
Corn 8% CP	755.503	809.513	855.971	896.058	766.560	818.199	864.545	902.689
Soybean meal 46%	205.000	149.000	100.000	58.000	194.000	140.000	91.000	51.000
NaCl	4.1780	3.936	3.433	3.431	4.177	3.934	3.432	3.430
Limestone	8.690	7.050	6.310	5.690	8.720	7.070	6.320	5.700
Phosphate	14.030	10.730	9.010	8.300	14.130	10.820	9.080	8.360
Vitamin/mineral Premix^1^	4.000	4.000	4.000	4.000	4.000	4.000	4.000	4.000
DL-Methionine	1.400	1.100	0.900	0.700	1.300	1.000	0.900	0.700
L-Lysine	4.500	4.500	4.500	4.500	4.500	4.500	4.500	4.500
L-Threonine	1.500	1.300	1.200	1.100	1.400	1.300	1.200	1.100
L-Tryptophan	0.600	0.600	0.650	0.650	0.600	0.600	0.650	0.650
L- Valine	0.600	0.600	0.600	0.600	0.600	0.500	0.600	0.600
Kaolin	-	7.670	13.425	16.970	0.012	8.076	13.772	17.270
Total	1,000.00	1.000.00	1.000.00	1.000.00	1.000.00	1.000.00	1.000.00	1.000.00
Calculated nutritional levels								
CP (%)	16.1590	14.0125	12.0935	10.4690	15.7342	13.6437	11.7752	10.1935
ME (Kcal/kg)	3.218.4680	3.220.0000	3.220.0000	3.220.0000	3.220.0000	3.220.0000	3.220.0000	3.220.0000
EE (%)	3.0678	3.2124	3.3328	3.3598	3.1124	3.2541	3.3723	3.4528
Ca (%)	0.7200	0.5700	0.4900	0.4400	0.7200	0.5700	0.4900	0.4400
P available (%)	0.3500	0.2800	0.2400	0.2200	0.3500	0.2800	0.2400	0.2200
Lys dig (%)	1.0155	0.8806	0.7647	0.6621	0.9888	0.8574	0.7446	0.6447
M. + C dig (%)	0.5994	0.5196	0.4588	0.3971	0.5836	0.5059	0.4467	0.3866
Tre dig (%)	0.6602	0.5728	0.4968	0.4303	0.6428	0.5577	0.4837	0.4190
Tryp dig (%)	0.2033	0.1757	0.1529	0.1320	0.1979	0.1711	0.1489	0.1285
Val dig (%)	0.7011	0.6080	0.5272	0.4569	0.6826	0.5920	0.5133	0.4449
Sodium (%)	0.1900	0.1800	0.1600	0.1600	0.1900	0.1800	0.1600	0.1600

^3^Vitamin/mineral premix supplies per kilogram of diet: vitamin A. 4500 UI; vitamin D3. 1000 UI; vitamin E. 20 mg; vitamin K3. 2 mg; vitamin B1. 0.9 mg; vitamins B2. 3 mg; vitamin B6. 1 mg; vitamin B12. 15 mcg; niacin. 12 mg; pantothenic acid. 10 mg; folic acid. 0.5 mg; biotin. 0.04 mg; choline. 400 mg; iron. 80 mg; cooper. 75 mg; manganese. 30 mg; zinc. 80 mg; iodine. 0.8 mg; and selenium. 0.25 mg. CP: Crude protein. EE: Ether extract. Ca: Calcium. P available: Available phosphorus. ME: Metabolizable energy. Lys dig.: Digestible lysine. M + C dig.: Digestible methionine + cysteine. Tre dig.: Digestible threonine. Tryp dig.: Digestible Tryptophan. Val dig.: Digestible valine.

The protease used in NC100 and NC150 is produced through the fermentation of *aspergillus niger*, with a concentration of 10.000 u/g.

### Experimental procedures

The feed and leftovers from the pens were collected and weighed daily. The animals were weighed on days 1, 26, 49, 75, and 104. Based on the data, performance was calculated as average daily weight gain (ADG), average daily feed intake (ADFI), feed conversion rate (FCR), and feed efficiency (FE), the animals received feed and water ad libitum during the experimental period*.*

On the third day preceding day 26 (second weighing of the animals), the animals received a diet corresponding to their assigned treatment, with a titanium oxide marker (TiO_2_) (dosage of 0.5%) for the digestibility analysis^13^.

On day 26 of the experiment, feces were collected from all animals via rectal stimulation for bromatological and digestibility analyses. The samples comprised a fecal pool for each pen and were frozen until laboratory analysis. This phase was selected with the objective of assessing nutrient digestibility in younger animals, considering those that might not have access to enzymes. It was assumed that the physiological adaptation of older animals would enhance endogenous digestibility.

The apparent digestibility coefficient (ADC) of dry matter (DM), mineral matter (MM), crude protein (CP), ether extract (EE), calcium (Ca), and phosphorus (P) were calculated as follows equation:

1 $${\text{ADC}}\left[ \% \right] = \left[ {{1} - \left( {{\text{TiO}}_{{2}} \,{\text{diet}}/{\text{TiO}}_{{2}} \,{\text{feces}}} \right) \times \left( {{\text{nutrient}}\,{\text{feces/nutrient}}\,{\text{diet}}} \right)} \right]^{{{13}}}$$

On the 104^th^ day of the experiment, a saliva sample was collected using an oral swab for cortisol analysis. One immobile barrow per pen were selected, randomly, without any restraint (in a delicate and precise manner), and the swab was offered to the animals for voluntary chewing, The samples were immediately placed in a polystyrene foam box containing dry ice at − 23 °C and sent to the laboratory.

Competitive ELISA using a commercial kit (Elabscience—Porcine Cortisol ELISA Kit – batch XPE5QZ3HPB. from United States) was used to quantify the cortisol levels. After saliva collection, blood was collected from eight barrows in each treatment, with a total of 40 animals employed for this analysis. The animals were randomly selected, with one animal chosen per pen. Samples were obtained from the jugular vein. Immediately after collection, the samples were transferred to sterile tubes containing EDTA (1 mg/mL) and tubes without anticoagulants to obtain whole blood and serum. Circulating cells, including red (erythrocytes, hemoglobin, hematocrit, and mean corpuscular volume) and white (leukocytes, neutrophils, lymphocytes, eosinophils, and monocytes) cells, were quantified using commercial kits, The hemogram and leukogram were analyzed using an Automated Hematology Analyzer Celltac MEK 6550– Nihon Kohden® (Tokyo, Japan). Quantitative analysis were performed to determine the levels of erythrocytes, hematocrit, hemoglobin, mean corpuscular volume (MCV), mean corpuscular hemoglobin concentration (MCHC), platelets, and total leukocyte count. For the differential analysis of leukocytes and morphological classification, a blood smear stained with Fast Panotic dye (Labor-clin®, São Paulo, Brazil) was viewed under an optical microscope.

One day before slaughter, the loin eye area and backfat thickness of all animals were measured using an ultrasound device (Aloka SSD-500, USA), and all assessments were performed by a qualified professional.

The animals were slaughtered on the 105th day of the experiment. The animals were subjected to a pre-slaughter fasting period of approximately 12 h and transported to a commercial slaughterhouse at night. Slaughtering was performed via electronarcosis followed by exsanguination. Body weight, carcass pH, and carcass weight were measured from the personnel at the slaughterhouse to calculate carcass yield. The carcass yield was calculated using the following equation:

2 $${\text{Carcass}}\,{\text{yield}}\,\left( \% \right) = \left( {\left( {{\text{hot}}\,{\text{carcass}}\,{\text{weight}}/{\text{body}}\,{\text{weight}}} \right) \times {100}} \right).$$

### Economic analysis

At the end of the experiment, an economic analysis was conducted to evaluate the feasibility of using the protease in the diets of swine in the growth and finishing phases. The variables were feed costs cost per kilogram of fattened swine produced, economic profit per fattened swine marketed and return on investment, according to data from the São Paulo Pig Production Cost Index (SPPPCI) for June 2021. The total production cost comprised (i) the cost of acquiring animals (33.8%). (ii) feed costs (51.3%), and (iii) other costs (14.9%). The item “other costs” comprised costs for labor, sanity, reproductive management, consumer goods, transport and insurance, Maintenance, depreciation, electricity and fuel, telephony and internet, fees and taxes, and opportunity costs of capital and land. For the revenue composition of the activity, the average sale value of the animal, as stipulated in the swine exchange of the Associação Paulista dos Criadores de Suínos (APCS) for June 2021 (US$ 1.46), was considered. To calculate the ROI, the following equation was used:

3 $${\text{ROI}} = {(}\left( {{\text{gross}}\,{\text{profit}} \times {\text{amount}}\,{\text{invested}}} \right)/{\text{amount}}\,{\text{ invested)}} \times {100}$$

### Statistical analysis

The Shapiro–Wilk test was performed to assess data abnormalities. When the data did not show abnormal distribution, data transformation was performed using PROC RANK (SAS INSTITUTE Inc. 2009).

All variables were subjected to analysis of variance (ANOVA). When a statistically significant difference was identified F-test (*P* < 0.05), Tukey’s test was performed to compare the means. Data were analyzed using the SAS software package, version 9.4 (2009) (www.sas.com) and the MIXED procedure.

### Ethics approval

All procedures involving animals were approved by the Ethics Committee in Use of Animals of the School of Veterinary Medicine and Animal Science. University of São Paulo (approval number CEUA 8428221220). Consent o participate is not applicable.

## Results

### Growth performance

The performance data are listed in Table 2.

**Table 2:** Performance of swine administered diets containing proteases in the growing and finishing phases.

Variables	Treatment^1^					CV %	SEM	*P* value
	PC	NC	NC100	NC150	NC200			
Initial BW. kg	25.56	25.50	25.56	25.61	25.57	14.90	1.331	0.671
1–26 days								
BW 26 days. kg	42.33^b^	47.35^a^	46.94^a^	46.32^a^	47.08^a^	13.44	2.058	< 0.0001
ADG	0.645^b^	0.840^a^	0.822^a^	0.796^a^	0.827^a^	16.16	0.036	0.0001
ADFI	1.618^b^	1.804^a^	1.751^ab^	1.747^ab^	1.769^ab^	14.93	0.088	0.015
FCR	2.461^a^	2.152^ab^	2.128^b^	2.082^b^	2.136^ab^	9.23	0.053	0.015
FE	0.404^b^	0.466^a^	0.472^a^	0.480^a^	0.469^ab^	9.50	0.012	< 0.0001
27–49 days								
BW 49 days. kg	63.71^b^	69.90^a^	72.90^a^	73.76^a^	72.95^a^	13.82	3.150	< 0.0001
ADG	0.929^c^	0.980^bc^	1.128^ab^	1.193^a^	1.124^ab^	18.12	0.058	< 0.0001
ADFI	2.412^b^	2.652^ab^	2.714^ab^	2.769^a^	2.736^a^	16.00	0.142	0.018
FCR	2.6^ab^	2.632^a^	2.401^c^	2.411^c^	2.433^bc^	6.56	0.048	0.001
FE	0.385^bc^	0.381^c^	0.417^a^	0.416^a^	0.411^ab^	6.50	0.008	0.001
1–49 days								
ADG	0.778^b^	0.906^a^	0.966^a^	0.982^a^	0.967^a^	15.15	0.041	< 0.0001
ADFI	1.991^b^	2.202^a^	2.203^a^	2.227^a^	2.223^a^	14.93	0.109	0.010
FCR	2.552^a^	2.425^ab^	2.276^b^	2.269^b^	2.296^b^	6.56	0.038	< 0.0001
FE	0.393^c^	0.413^bc^	0.44^a^	0.441^a^	0.435^ab^	6.42	0.007	< 0.0001
50–75 days								
BW 75 days	89.979^b^	97.398^ab^	100.99^a^	103.01^a^	101.27^a^	12.68	4.047	< 0.0001
ADG	1.010	1.058	1.080	1.105	1.089	15.46	0.057	0.758
ADFI	3.009	3.154	3.233	3.190	3.238	14.86	0.162	0.559
FCR	2.899	3.009	3.027	2.875	2.971	10.04	0.108	0.789
FE	0.335	0.335	0.333	0.352	0.338	10.48	0.012	0.812
1–75 days								
ADG	0.867^b^	0.958^ab^	1.005^a^	1.025^a^	1.009^a^	12.41	0.041	0.001
ADFI	2.344^b^	2.532^ab^	2.560^ab^	2.561^ab^	2.575^a^	13.67	0.116	0.046
FCR	2.675^a^	2.637^ab^	2.545^bc^	2.490^c^	2.545^bc^	4.95	0.039	< 0.0001
FE	0.374^c^	0.380^bc^	0.393^ab^	0.402^a^	0.393^ab^	4.89	0.006	< 0.0001
75–104 days								
BW 104 days	128.65	131.96	134.56	136.64	137.45	9.08	4.335	0.062
ADG	1.312	1.192	1.158	1.160	1.245	12.80	0.052	0.057
ADFI	3.597	3.723	3.587	3.637	3.826	13.71	0.179	0.597
FCR	2.983	3.118	3.099	3.207	3.129	8.72	0.105	0.673
FE	0.336	0.321	0.324	0.315	0.322	8.34	0.010	0.715
1–104 days								
ADG	0.993	1.023	1.048	1.062	1.082	9.23	0.034	0.087
ADFI	2.693	2.864	2.846	2.865	2.941	12.73	0.122	0.143
FCR	2.771	2.795	2.748	2.694	2.718	4.17	0.045	0.312
FE	0.361	0.358	0.364	0.372	0.368	4.26	0.006	0.311

SEM: Mean standard error. BW: Body weight. ADG: Average daily gain. ADFI: Average daily feed intake. FCR: Feed conversion ratio. FE: Feed efficiency. Averages followed by different lowercase letters in a row differ according to Tukey's test, with *P* < 0.05.

On the first period (day 1–26) animals in the PC group had significant lower (average − 27.32%) ADG and consequently significant lower BW (average − 10.84%) compared to the animals in the other groups (*P* < 0.001) and ADFI was significant lower (average − 10.31%) compared to NC (*P* < 0.0015). FCR was considerable better (average − 14.63%), just like FE (average 15.12%) for animals in the NC100 and NC150 groups than animals in the PC group.

In the second experimental period (day 27–49). animals in the PC and NC groups had considerably lower (PC − 29.11% and NC − 21.73%) ADG than animals in the NC150 group (*P* < 0.0001); however, only animals in the PC group had a lower weight on day 49 (− 13.60%) (*P* < 0.0001). The animals treated with NC100 and NC200 had substantial lower feed conversion (average -7.46%) than those treated with PC and NC (*P* = 0.001).

In the third period (day 50–75), only the BW on day 75 (BW75) differed between the experimental groups. Where animals in the PC group had lower BW75 (average − 0.13%) than those supplemented with proteases (*P* < 0.0001). During the final experimental period (day 76–104). No treatment effect was observed (*P* > 0.05).

During the entire experimental period (1–104 days). the treatments were not observed to significantly affect the performance of animals (*P* > 0.05).

### Carcass characteristics

The carcass characteristics of animals are shown in Table 3.

**Table 3:** Carcass characteristics of swine administered diets containing proteases in the growing and finishing phases.

Variables	Treatments^1^					SEM	*P* value
	PC	NC	NC100	NC150	NC200		
Carcass weight (kg)	101.01^b^	105.58^ab^	107.79^ab^	108.94^a^	110.09^a^	3.889	0.009
Carcass yield (%)	79.03	80.07	80.49	79.78	80.08	0.41	0.075
Backfat thickness (mm)	16.65	18.6	17.92	19.42	18.64	1.303	0.265
Loin eye area (cm^2^)	41.48^b^	42.17^ab^	45.71^ab^	46.11^a^	45.66^ab^	1.593	0.021
CCMY (%)	52.03	50.66	51.00	49.93	50.41	0.874	0.167
Bonus index	103.92	104.42	105.41	104.68	105.46	0.663	0.228
Initial pH Carcass	6.15	6.19	6.15	6.14	6.16	0.044	0.824

SEM: Mean standard error. CCMY: chilled carcass meat yield. Averages followed by different lowercase letters in a row differ according to Tukey's test, with *P* < 0.05.

Animals in the PC group had significant lower carcass weights than those in the NC150 (− 7.85%) and NC200 (− 8.98%) groups (*P* = 0.009). Furthermore, animals in the NC150 group had significant bigger (11.46%) loin eye area than those in the PC group (*P* = 0.021).

### Digestibility

Table 4 shows the apparent digestibility coefficients (DC) of different nutrients.

**Table 4:** Apparent digestibility coefficients of nutrients in the diet of swine administered diets containing proteases in the growing and finishing phases.

Variables %	Treatments^1^					SEM	*P* Value
	PC	NC	NC100	NC150	NC200		
DCDM	73.67^b^	72.35^b^	77.87^a^	75.56 ^ab^	73.36b	0.9333	0.001
DCMM	32.95^b^	39.82^a^	31.04^b^	27.10^b^	28.69^b^	2.0654	0.0001
DCCP	52.49^b^	66.18^a^	64.78^a^	62.76^a^	60.27^a^	1.6634	< 0.0001
DCEE	62.76^a^	59.31^ab^	54.85^ab^	51.42^b^	51.42^b^	2.1647	0.002
DCCa	30.98^ab^	40.39^a^	29.33^b^	31.31^ab^	25.44^b^	3.3077	0.0074
DCP	49.10^b^	54.72^a^	47.23^ab^	27.22^c^	26.17^c^	2.5407	< 0.0001

SEM: Mean standard error. DCDM: apparent digestibility coefficient of dry matter. DCMM: apparent digestibility coefficient of mineral matter. DCCP: digestibility coefficient of crude protein. DCEE%: digestibility coefficient of the ether extract. DCCa: apparent digestibility coefficient of calcium. DCP: apparent digestibility coefficient of phosphorus. Averages followed by different lowercase letters on a line differ significantly according to Tukey's test with *P* < 0.05.

The DCDM was significant superior for animals receiving the NC100 diet compared to animals administered the PC (5.7%)**,** NC (7.62%)**,** and NC200 (1.67%) diets (*P* = 0.001). DCMM was substantial superior (24.79%) in the NC group compared to the other experimental groups (*P* = 0.0001). The PC group had considerable lower DCCP (average -21.16%) than the other groups (*P* < 0.0001); for DCEE**,** animals in the NC150 and NC200 groups had substantial lower (-17.95%) digestibility than those in the PC group (*P* = 0.002). For calcium**,** a higher DC was observed in animals receiving the NC diet than in those receiving the NC100 (37.70%) and NC200 (58.76%) diets (*P* = 0.0074). Similarly**,** for DCP**,** significant greater digestibility was observed in animals receiving the NC diet than in animals administered the PC (36.455)**,** NC150 (101.02%)**,** and NC200 (108.29%) diets (*P* < 0.0001).

### Economic viability

Table 5 shows the economic analysis results of using protease in the diets of swine in the growth and finishing phases.

**Table 5:** Economic analysis of the production of swine administered diets containing proteases in the growing and finishing phases.

Variables	Treatments^1^					SEM	P. Value
	PC	NC	NC100	NC150	NC200		
Feed cost R$	496.48	524.63	522.87	518.29	532.58	23.66	0.424
Cost per kg of animal produced R$	8.19^a^	8.09^ab^	7.93^ab^	7.76^b^	7.76^b^	0.16	0.019
Profit per fattened swine R$	84.81	89.85	102.67	121.94	118.63	11.05	0.086
ROI	10.3157	10.7176	12.3922	14.7582	14.4366	1.72	0.163

SEM: Mean standard error. ROI: Return on Investment. Averages followed by different lowercase letters in a line differ significantly according to Tukey's test with *P* < 0.05.

Treatment did not affect feed costs, fattened swine profits, or return on investment (ROI) (*P* > 0.05). However, animals in the NC150 and NC200 groups had a significant lower cost per kilogram of animals produced than the PC group (*P* = 0.019).

### Blood parameters and salivary cortisol

The blood parameters and salivary cortisol levels did not statistically differ (*P* > 0.05) among the groups (Table 6).

**Table 6:** Blood count and salivary cortisol level in swine administered diets containing proteases in the growing and finishing phases.

Variables	Treatmens^1^					SEM	*P* value
	PC	NC	NC100	NC150	NC200		
Red cells (millions/mm^3^)	7.6112	7.095	6.81	7.2833	6.9871	0.1077	0.1378
Hemoglobin (g/dl)	13.425	12.3375	12.075	12.8333	12.4857	0.1700	0.0804
Hematocrit (%)	43.6875	40.1625	39.3125	42.56	41.1857	0.9656	0.0895
MCV (fl)	57.3375	56.6625	57.7375	58.0167	59.2429	0.4224	0.416
MCH (pgc)	17.625	17.4	17.7375	17.6667	17.9714	0.1328	0.7663
MCHC (g/dl)	30.7375	30.725	30.7	30.4167	30.3429	0.070	0.2394
Monocytes Log^10 (%)	591	668.857	630.429	638.833	448.167	64.9305	0.641
Eosinophils (%)	3451.75	3579.88	3700.13	2465	2525.14	258.0.49	0.4083
Neutrophils (%)	9404.13	12,271.8	10,853.5	14,171.7	9764.43	564.431	0.0589
Platelets (103/ul)	288,625	238,875	261,000	223,333	264,857	12,557.43	0.5658
Total Lymphocytes (1000/mm^3^)	19,700	22,525	22,312.5	23,816.7	18,828.6	627.8704	0.1350
Lymphocytes (%)	6263.88	6088.13	7148	6541.17	6127.86	269.6446	0.2013
Cortisol root	1.608	1.575	1.4157	1.233	1.481	0.453	0.984

SEM: Mean standard error. MCV: Mean corpuscular volume. MCH: Mean corpuscular hemoglobin. MCHC: Mean corpuscular hemoglobin concentration. Monocytes Log^10^: Log^10^ of monocyte concentration. Cortisol Root: Square root of mean salivary cortisol.

## Discussion

Contrary to expectations, the NC showed better results when compared to the PC. The initial hypothesis would suggest NC would have an inferior result compared to all treatments, which in practice did not happen.

It is possible to observe that in the first period of the experiment, 1–26 days, the NC animals showed higher consumption when compared to the PC animals, however, there were no differences between the other treatments. This is the key to understanding the better performance achieved by the NC in relation to the PC. Although without statistical difference, Fang at, al.^14^ also observed an increase in ADFI (200 g/animal) with a decrease in dietary CP, especially in the first weeks.

The first period impacts up to 75 days of experiment, however, evaluating in a stratified manner, the ADFI will be equal from the second period between NC and PC.

In general, animals that received protease-containing diets exhibited better performance, corroborating the results previously described^15–18^ and led to better economic returns than those that did not receive the enzyme. The prerogative of using technologies in animal production presupposes gains in this sense, which is key in decision-making regarding the inclusion of additives.

Given the increasing expenses associated with protein sources like soybean meal (SBM), significant efforts are being made to lower the protein content of animal feed while ensuring optimal animal performance^2^. This concept entails assigning a matrix value to amino acids for the exogenous protease, thereby decreasing the amino acid concentration in the diet^19^. In this study, a matrix comprising 2.5% CP and amino acids was suggested. The probable reason for treatments outperforming controls is likely an underestimation.

In contrast to this study, a meta-analysis of 67 experiments^4^ did not reveal positive results with the inclusion of proteases in a monogastric diet. Other authors^20,21^ also obtained results that oppose those of the present study. Unlike other enzymes, proteases are more specific, and the basic concepts of substrate and enzyme specificity must be considered when choosing the type of protease.

In the present study, an acidic protease belonging to the aspartate protease family, and a neutral protease belonging to the serine protease family was employed. The type and, therefore, low specificity like endogenous proteases, of the exogenous protease used may be key to obtaining positive results.

Another relevant point evidenced in this work, when stratified by growing versus finishing phase, is that the technology applied to younger animals apparently resulted in a better action based on evaluations performed in the later stages of production^22,23^. Although a difference was observed between treatments in BW at 75 days, this difference was justified by the accumulated gains of the previous phases, however, ADG did not differ after 50 days of housing.

As no statistical difference in productive performance was found, animals displayed better physiological adaptation to the use of nutrients from the diet in the last phase. However, when the entire period was compared, the use of proteases resulted in the best economic return (Profit and ROI).

The DCDM increased in animals administered acid proteases (NC100 and NC150). These results are consistent with those of previous studies on nursery piglets^24–26^. However, in terms of DCCP, the NC group did not differ from the treatment groups administered protease, unlike the other results^25^. The researchers observed reduced crude protein digestibility in piglet diets with decreased soybean meal content, based on these results, CP digestibility can be hypothesized to differ according to animal age, even after the nursery period, a physiological adaptation may exist in which digestive metabolism alone increases protein digestion from lower concentrations in the diet, corroborating the results of Choe et al.^16^, who observed less effectiveness in the use of proteases in the finishing phase owing to the development of the digestive tract.

The use of proteases favored better performance, and due to greater nitrogen digestibility, greater muscle protein retention was obtained^27^, and improvement in the carcass^28,29^. Similar results were obtained in this study, where NC150 resulted in a greater loin eye area and higher DCCP than PC, which leads to the assumption that the increase in digestibility and therefore greater quantity of amino acids absorbed culminates in better carcass characteristics. In contrast, other authors^16,17^, did not find benefits to the carcass with the addition of proteases to pig diets.

In addition to the zootechnical results, the health and welfare of animals subjected to new technologies must be examined. Hematological studies can be used to monitor food stress^30^. Blood analysis enables the evaluation of various metabolites and other components within an animal's body, which are crucial factors in determining the physiological^31^, nutritional and pathological conditions of an organism^32,33^ and their relationship with the environment^34,35^.

The inclusion of protease in the diets did not affec the blood parameters of the animals. Furthermore, all values were within the limits established for the species^36,37^.

Similarly, stressors can have negative effects on growth and consequently, animal health^38,39^. Health is one of the pillars of animal welfare, therefore, understanding stress status from a physiological point of view is important for animal production. One of the best approaches to assess the health and welfare status of swine using non-invasive and easy methods involves the analysis of cortisol from saliva samples^40^, using a technique previously validated^41^.

Cortisol assessment is important because proteins can alter serum cortisol levels. The amount of protein in the diet directly affects the salivary cortisol levels in pigs^11^. These researchers evaluated the effects of different protein levels in pig diets on salivary cortisol levels and found that excessive protein intake significantly increased the salivary cortisol levels. Furthermore, increased levels of salivary cortisol are associated with increased aspartate aminotransferase activity, suggesting a relationship between stress and liver function in pigs. However, salivary cortisol level did not differ among the treatment groups, suggesting that the decrease in protein and the inclusion of protease did not generate stressful factors from a nutritional point of view.

Finally, the rearing of swine for meat distribution to consumers should be examined, as performed for other businesses. Understanding the production costs and profits is essential to production viability. When the cost of a soybean meal is high, alternatives that reduce inclusion or improve utilization are even more viable. The inclusion of proteases in this study, regardless of the source and dose, improved production profitability compared with diet without protease. In addition, a better ROI was obtained when proteases were included in the diet.

## Conclusion

The use of aspartic protease at both dosages, 100 g/mT and 150 g/mT. especially the latter, in a diet with reduced crude protein and amino acids can be recommended to reduce production costs, increase animal performance thereby improving the profitability and viability of swine production. Research examining the dietary impact of protein variations holds promise for elucidating the physiological adaptations in animal metabolism. Furthermore, we propose investigations into the metabolic capacity to generate endopeptidases through exogenous proteases.

## Data Availability

The datasets used and analyzed during the current study are available from the corresponding author on reasonable request.
